# Chemical Vapour Deposition of MWCNT on Silica Coated Fe_3_O_4_ and Use of Response Surface Methodology for Optimizing the Extraction of Organophosphorus Pesticides from Water

**DOI:** 10.1155/2019/4564709

**Published:** 2019-07-03

**Authors:** Veronica W. O. Wanjeri, Sefater Gbashi, Jane C. Ngila, Patrick Njobeh, Messai A. Mamo, Patrick G. Ndungu

**Affiliations:** ^1^University of Johannesburg, Department of Chemical Science, Energy, Sensors and Multifunctional Nanomaterials Research Group, P.O Box 17011, Doornfontein 2028, South Africa; ^2^Department of Biotechnology and Food Technology, University of Johannesburg, South Africa

## Abstract

Multiwalled carbon nanotube (MWCNT) was fixed onto the surface of a magnetic silica (Fe_3_O_4_@SiO_2_) substrate via chemical vapour deposition (CVD). Acetylene gas was used as the carbon source and cobalt oxide as the catalyst. The chemical and physical characteristics of the materials were investigated by transmission electron microscopy (TEM), Raman spectroscopy (RS), scanning electron microscopy (SEM), energy-dispersive spectroscopy (EDS), X-ray diffraction (XRD), and nitrogen adsorption/desorption isotherm. The synthesized Fe_3_O_4_@SiO_2_-MWCNT nanocomposite was used as a magnetic solid phase extraction (MSPE) adsorbent for the preconcentration of organophosphorus pesticides (OPPs), specifically, azinphos methyl, chlorpyrifos, parathion, and malathion. The factors influencing the extraction efficiency such as pH, contact time, and adsorbent dosage were investigated and optimized by response surface methodology (RSM) and desirability function. Linear response was obtained in the concentration range of 10–200 *μ*g/L for the analytes with determination coefficients ranging between 0.9955 and 0.9977. The limits of detection (LODs) and quantification (LOQs) were in the range of 0.004-0.150 *μ*g/L and 0.013-0.499 *μ*g/L, respectively. Fe_3_O_4_@SiO_2_-MWCNT was applied in the extraction and subsequent determination of OPPs in water samples from Vaal River and Vaal Dam with recoveries ranging from 84.0 to 101.4% (RSDs = 3.8–9.6%, n = 3) in Vaal River and 86.2 to 93.8% (RSDs = 2.9–10.4%, n = 3) in Vaal Dam. The obtained results showed that the newly synthesized Fe_3_O_4_@SiO_2_-MWCNT nanocomposite can be an efficient adsorbent with good potential for the preconcentration and extraction of selected OPPs from aqueous media.

## 1. Introduction

Pesticides are widely used in agriculture because they are highly effective in controlling and eradicating insect, pests, and weeds. This enhances crop yield and results in high-quality product [1]. However, extensive use of these substances in agriculture eventually leads to long term accumulation and some hazardous effects to ecosystems and human health [2, 3]. In recent years, organophosphorus pesticides (OPPs) have been widely used in agriculture due to their high efficiency as insecticides. However, their slow degradation and extensive or inappropriate use by farmers can lead to their residues present in the environment, including water, soil, and agricultural products [4, 5]. OPPs tend to pose adverse risks to nontarget organisms and humans, as they are known to be neurotoxic and immunotoxic and equally affect reproduction [6, 7]. Contamination of surface water and ground water by OPPs is a global concern and continues to be an active area of research. European Union Directive on drinking water quality (98/83/EC) has established a maximum allowed concentration of 0.1 ng/mL for each individual pesticide and 0.5 ng/mL for total pesticides [8–10]. Thus, to help protect human and animal health and the aquatic environment against these substances, determination of trace OPPs in environmental samples is of tremendous importance.

MWCNTs have contributed significantly to the field of nanoscience especially in adsorption and separation sciences, due to their unique tubular structures, large length-to-diameter ratio, and their excellent chemical-physical properties [11]. Several methods have been adopted in the synthesis of MWCNTs, including arc discharge [12, 13], laser ablation [12], and chemical vapour deposition (CVD) [12–14]. The CVD method is relatively simple and versatile, produces high-quality product at a relatively low cost, and enables direct growth of CNTs onto substrates; hence it is widely used. Another significant advantage with CVD is that the catalyst can be held on a substrate, which can lead to the growth of vertically aligned CNTs (VACNTs) in a desired direction with respect to the substrate [14, 15]. Typically, transition metal catalysts such as Ni, Fe, Co, and their alloys are used in the synthesis of MWCNTs, and this is due to the significant high yield that can be obtained with such catalysts [16].

For the extraction and preconcentration of OPPs from complex matrices, to enhance sensitivity and selectivity, liquid-liquid extraction (LLE) and solid phase extraction (SPE) are the most commonly used sample preparation techniques [17]. However, LLE has some disadvantages such as long work-up times and use of large amounts of relatively toxic organic solvents [4, 18]. SPE is one of the most commonly used techniques in the extraction and preconcentration of various compounds and elements from environmental samples due to its high recovery, short extraction time, high enrichment factor, low use of organic solvents, and ease of automation of the whole process. Despite these advantages, the technique has some drawbacks, including high back pressure in the packing process and low extraction efficiencies and it can be relatively expensive depending on the type of SPE applied [18, 19]. Some of the SPE methods that have recently been developed to overcome these problems include solid phase microextraction (SPME) [20], dispersive solid phase extraction (DSPE) [21], magnetic solid phase extraction (MSPE), molecularly imprinted solid phase extraction (MISPE), and matrix solid phase dispersion extraction (MSPDE) [19].

Among these techniques, MSPE has proven to be an interesting procedure in which the analytes are adsorbed onto the magnetic adsorbent and then the adsorbent together with the adsorbed analytes is separated from the sample solution using an external magnetic field. The analytes are finally eluted from the adsorbent and analyzed [18]. The advantages of MSPE include prevention of problems related to column packing, analyte can be separated from a large sample volume, and separation process can be performed directly in crude samples containing suspended solids without the need for additional centrifugation or filtration, which makes the separation easier and faster [19, 22]. Magnetite Fe_3_O_4_ is one of the most widely used magnetic materials because it presents a high surface area and excellent magnetic properties, being less toxic and easy to synthesize and functionalize [23]. Unprotected Fe_3_O_4_ nanoparticles easily aggregate, react with O_2_ present in the air, and can degrade organic compounds in aqueous systems. To prevent such limitations, Fe_3_O_4_ nanoparticles can be coated with a protective layer of different materials such as silica, polymer, noble metals, and carbon nanomaterials, improving their stability and introducing new surface properties and functionalities [24]. Silica is stable at high temperatures, especially at temperatures used for CVD growth of MWCNTs, and thus was used to coat Fe_3_O_4_ nanoparticles in the current study. In addition, the silica can shield or limit the magnetic interaction between the Fe_3_O_4_ nanoparticles, and since the silica shells are negatively charged [25], this can further reduce the aggregation of the nanoparticles.

Previously, we studied the adsorption isotherms of chlorpyrifos, parathion, and malathion on a magnetic graphene oxide composite [10]. In this study, MWCNTs were synthesized on a magnetic silica (Fe_3_O_4_@SiO_2_) substrate using a CVD technique, with cobalt oxide as a catalyst and acetylene gas as the carbon source. The prepared Fe_3_O_4_@SiO_2_-MWCNT was characterized using different analytical techniques and applied in the extraction of selected OPPs (supplemental data, Figure S1) from water samples. Various factors affecting the extraction efficiency including pH, contact time, and adsorbent dosage were optimized using response surface model based on central composite design (CCD) combined with desirability function (DF). The optimum conditions were then utilized for the MSPE of OPPs from environmental water samples collected from Vaal River and Vaal Dam in South Africa.

## 2. Materials and Methods

### 2.1. Chemicals and Reagents

All chemicals used in this work were of analytical grade. Potassium permanganate, hydrogen peroxide, iron (II) chloride (FeCl_2_·4H_2_O), hydrazine, tetraethylorthosilicate (TEOS), and cobalt (II) nitrate hexahydrate were obtained from Sigma–Aldrich Ltd. (South Africa). Ethanol (EtOH), iron (III) chloride (FeCl_3_·6H_2_O), and acetone were purchased from Merck Chemicals (South Africa). Acetylene gas and nitrogen gas were obtained from Afrox (South Africa).

Pesticide analytical standards (azinphos methyl, chlorpyrifos, parathion, and malathion) were purchased from Sigma–Aldrich Ltd. (South Africa). All pesticide standards were of 98–99% purity. Stock solutions of each compound with a concentration of 200 mg/L were prepared in HPLC-grade acetonitrile. Working standards solutions were prepared by diluting the stock solutions to appropriate concentrations in acetone. The stock and working standards were all stored at 0°C.

### 2.2. Synthesis of Fe_*3*_O_*4*_ and Fe_*3*_O_*4*_@SiO_*2*_

The synthesis of the iron oxide nanoparticles and subsequent coating with silica were done using methods we have previously reported [10]. In a typical synthesis procedure, 5 mL of ammonia, 2 mL of hydrazine, and 50 mL of deionized water were mixed in a 100 mL two neck round bottom flask. Then 20 mL of an aqueous solution of 1.0 g of FeCl_2_*∙*4H_2_O and 2.7 g of FeCl_3_*∙*6H_2_O was added dropwise. The solution was then placed in a preheated oil bath (90°C) and stirred for 60 minutes. After cooling to room temperature, the solid products were separated from the liquid using an external magnetic and then washed three times with water and acetone. The product was dried at 70°C for 12 hr and then weighed (approximately 3 g was produced).

Using methods we have previously optimized [10], the Fe_3_O_4_@SiO_2_ was prepared by adding 0.5 g of the Fe_3_O_4_ nanoparticles (NPs) to a glass bottle with a solution of 100 mL of ethanol and water (1:1, v/v) and 5 mL of 25% NH_4_OH solution. The bottle was capped (PTFE cap); the mixture sonicated for 30 minutes; and then 2 mL of TEOS was added. After stirring the mixture for 20 hours at room temperature, an external magnet was used to recover, the Fe_3_O_4_@SiO_2_ NPs. The sample was then repeatedly washed with water and ethanol, placed in an oven at 70°C, and allowed to dry for 24 hr (approximately 1 g was produced). A schematic illustration of the steps used to synthesize the Fe_3_O_4_@SiO_2_ nanoparticles is provided in the supplemental data (Figure S2).

### 2.3. Synthesis of MWCNT by CVD Process

Some of our previous work has used cobalt as a catalyst for the synthesis of CNTs [26]; thus the synthesis of MWCNT was by chemical vapour deposition (CVD) with cobalt oxide as catalyst. The prepared Fe_3_O_4_@SiO_2_ nanocomposite was seeded with a layer of cobalt oxide by dissolving 5.0 g cobalt (II) nitrate hexahydrate in 100 mL of methanol in a beaker. Then 1.0 g of Fe_3_O_4_@SiO_2_ was added to the solution. This mixture was stirred for 2* hr* at room temperature and then the solvent was evaporated on a vacuum rotary evaporator (IKA RV 10 basic) and dried at 200°C in an oven for 2* hr*. The final product was calcined at 600°C in a furnace for 6 hr. Then 0.2 g of the calcined substrate with catalyst was loaded into a quartz boat and placed inside a quartz tube reactor (100 cm × 4 cm i.d.). The quartz tube was then transferred to a horizontally aligned tube furnace (Lenton elite thermal limited) as shown in Figure S3. The CVD system was appropriately sealed, and the furnace temperature was ramped up to 800°C at 25°C/min under N_2_ gas at a flow rate of 500 mL/min. Once the temperature was stabilised at 800°C, N_2_ gas flow rate was set at 100 mL/min and acetylene gas at 300 mL/min was introduced into the reactor. After 60 min of reaction time, the acetylene gas was switched off and the system left to cool down to room temperature under a continuous flow of N_2_ gas at a flow rate of 50 mL/min. Finally, the quartz boat was then removed from the reactor and the synthesized MWCNTs on the substrate formed were weighed. The average mass of MWCNT formed was approximately 1 g.

### 2.4. Oxidation of MWCNTs

Oxidation of the synthesized Fe_3_O_4_@SiO_2_-MWCNT was done to purify and functionalize the surface of MWCNT using 30% HNO_3_ for 3* hr* at room temperature. Then the unreacted acid solution was removed, and MWCNTs were washed with deionized water until a pH of 7 was reached. The oxidised MWCNTs were dried at 70°C in an oven for 12 hr.

### 2.5. Characterization

The structural composition, morphological features, and physiochemical properties of the synthesized Fe_3_O_4_ NPs and the various nanocomposites (Fe_3_O_4_@SiO_2_ and Fe_3_O_4_@SiO_2_-MWCNT) were ascertained using different characterization techniques. Transmission electron microscopy (TEM) was done using a JEOL JEM-2100F Field Emission Electron microscope instrument equipped with a Lab6 source at an accelerating voltage of 200 kV. The images were captured using Gatan Orius CCD camera controller. Samples used for TEM analysis were prepared by dispersing the NP powder in ethanol followed by ultrasonication for 10 min. A drop of the dispersion was placed onto coated copper grid (200 mesh size Cu-grid). SEM-EDX was used to determine the element composition of Fe_3_O_4_@SiO_2_-MWCNT nanocomposite using Tescan VEGA 3 XMU scanning electron microscopy (TESCAN, Czech Republic). The nanoparticles were placed on the sample holder with carbon tape coated with gold nanoparticles and the images were taken using Vegas software. X-ray diffraction (XRD) was determined with a PANalytical X'Pert PRO X-ray diffractometer using Cu K*α* radiation set at a wavelength of 1.5406 Å at 40 kV and 40 mA in a range of 4-90° of 2 *θ* at room temperature. Nitrogen adsorption/desorption isotherm analysis was performed to determine the BET surface area, pore volume, and the average pore diameter were assessed according to the Barret–Joyner–Halenda (BJH) using a Micrometrics ASAP 2020 (Atlanta, Georgia, USA)

### 2.6. Extraction of Selected Pesticides Using Fe_*3*_O_*4*_@SiO_*2*_-MWCNT Adsorbent

Azinphos methyl, chlorpyrifos, parathion, and malathion (OPPs) in 20 mL aqueous solutions were extracted using Fe_3_O_4_@SiO_2_-MWCNT adsorbent in 100 mL Erlenmeyer flasks, which were kept at 25°C in a thermostatic water-bath shaker with a speed of 120 rpm. Thereafter, a magnet (NdFeB permanent external magnet purchased from Sable Magnets CC, 35 mm x 50 mm) was placed on the outside bottom of the Erlenmeyer flask to collect the adsorbent, and the supernatant discarded. Thereafter, the analytes were desorbed from the particles by vortexing the adsorbent with 2 mL of acetone (x3 and *∽*30 second each time). The desorbed solutions were added together and then the solvent was evaporated to dryness under nitrogen gas stream. The residue was redissolved in 5 mL acetonitrile and finally 2.0 *μ*L of the content injected into a LC-MS for analysis.

### 2.7. Determination of Organophosphorus Pesticides

Chromatographic separation and MS determination of OPPs were achieved using a Shimadzu LCMS 8030 equipment (Shimadzu Corporation, Tokyo, Japan), which is essentially an UHPLC instrument capable of obtaining 500 MRMs/sec, with an ultrafast scan speed of 15,000 u/sec, and a polarity switching of 15 m/sec. The liquid chromatography instrument was a LC-30AD Nexera connected to a SIL-30 AC Nexera autosampler and a CTO-20 AC Prominence column oven. The oven was equipped with a Raptor™ ARC-18 column from Restek (2.7 *μ*m, 2.1 mm × 100 mm) (Restek Corporation, Pennsylvania USA) and maintained at a constant temperature of 40°C. The mobile phases used consisted of Solvent A (0.1% formic acid in deionized water) and Solvent B (0.1% formic acid in acetonitrile), which was delivered at a constant flow rate of 300 *μ*L/min. The elution gradient program had a total run time of 11 min and started with 20% Solvent B for 2 min, increased steadily to 40% 2 min, and ramped to 95% in 2.5 min, at which point where it was kept constant for 2 min, and then the initial conditions (20% B) were reestablished for 1 min, and the column was allowed to reequilibrate for 2.5 min for the next run.

Following the chromatographic separation, analytes were committed to a Shimadzu triplequad mass spectrometry detector model 8030 (Shimadzu Corporation, Kyoto, Japan) for their detection and quantitation. The ionization source was an electron spray ionization (ESI) operated in a positive mode at an event time of 0.206 sec. Data was acquired by a multiple reaction monitoring (MRM) method at optimized MS conditions for the analytes (Table S1). The interface nebulizing gas flow rate was 3 L/min, DL temperature was 250°C, heat block temperature was 400°C, and drying gas flow rate was 15 L/min.

### 2.8. Experimental Design and Optimization of RSM

Optimization of factors affecting the* Fe*_*3*_*O*_*4*_*@SiO*_*2*_*-MWCNT nanocomposite* in the extraction of OPPs from water was achieved by means of response surface methodology (RSM) design of experiments (DoE) specifically by use of central composite design (CCD). RSM approach was adopted for three experimental factors (viz.,* pH, adsorbent dosage, and adsorbent time*) and one response variable (% extraction recovery) using Statistica version 8 (StatSoft, USA). This was carried out* using* a thermostatic water-bath shaker at 25°C at 120 rpm. The concentration of OPPs was kept at 50 *μ*g/L during the optimization. By using CCD, a total of 16 experimental runs (Table S2) were designed and the mathematical relationship between the three independent variables, pH (X_1_), adsorbent dosage (X_2_), and time (X_3_), and the dependent variable and % extraction recovery (Y) was approximated by using a 2^nd^ order polynomial model as presented in (1). In using this equation, linear (X_1_, X_2_, X_3_), quadratic (X_1_^2^, X_2_^2^, X_3_^2^), and interactive (X_1_X_2_, X_1_X_3_, X_2_X_3_) effects of independent variables were determined [5, 27, 28].

(1) $$\begin{array}{c} Y=\beta_{O}+\overset{3}{\underset{I=1}{\sum }}\beta_{i}X_{I}+\overset{3}{\underset{i=1}{\sum }}\beta_{ii}X_{i}^{2}+\overset{2}{\underset{i=1}{\sum }}\overset{3}{\underset{j=i+1}{\sum }}\beta_{ij}X_{i}X_{j} \end{array}$$

where Y is the predicted response; *β*_0_ the constant (intercept); *β*_i_ the linear coefficient; *β*_ii_ the quadratic coefficient; and *β*_ij_ the cross product coefficient. X_i_ and X_j_ are independent variables.

### 2.9. Desirability Function (DF)

Desirability function (DF) was used to determine the input variables that can give the optimal conditions for one or more responses based on Derringer's desirability function as described by Roosta et al., 2014 [29]. DF was used in the transformation of each predicted (${\hat{U}}_{i}$) and experimental response (Ui) to generate individual response (*d*_*i*_). The determined global function (D) should be maximum following selection of optimum value of the variables considering their interaction. The response (U) was first converted into a specific DF (*df*_*i*_) in the range between d=0, for complete undesirability response, to d=1 for a fully desired response above which further improvements would have no importance. With the individual desirability scores d_*i*_s, the combined desirability was obtained by using geometrical mean on single overall desirability (D), to establish the optimum set of input variables [29, 30].

(2) DF=df1υ2xdf2υ2⋯xdf2υ21/n,0≤υi≤1  i=1,2,…,n

where *df*_*i*_ indicate the desirability of the response U*i*  (*i* = 1,2, 3,…, *n*) and *υ*_i_ represents the importance of responses.

### 2.10. Environmental Water Sampling and Analysis

Environmental water samples were collected from Vaal River (-26.873045, 28.117264) and Vaal Dam (-26.851341, 28.146690) in the Gauteng province of South Africa. Water samples were collected in 2.5 L amber bottles previously washed with hot water and phosphate free detergent and further rinsed three times with deionized water. Sampling bottles were rinsed three times with the river and dam water prior to sample collection. After sampling, the bottles were closed with caps lined with aluminium foil to prevent contamination with phthalates and plasticizers from the lids. All samples were maintained in a cooler box containing ice and transported to the laboratory where they were kept in a fridge at 4°C prior to analysis. The water samples were filtered through a 0.45 mm filter paper prior to analysis using the optimum conditions obtained.

## 3. Results and Discussion

### 3.1. Characterization of the Adsorbent

The synthesized Fe_3_O_4_@SiO_2_-MWCNT was intensively characterized using different analytical techniques such as TEM, SEM, XRD, and N_2_ adsorption/desorption to confirm the physical, chemical and morphological properties.

#### 3.1.1. TEM and SEM Observations

**Figure 1:**
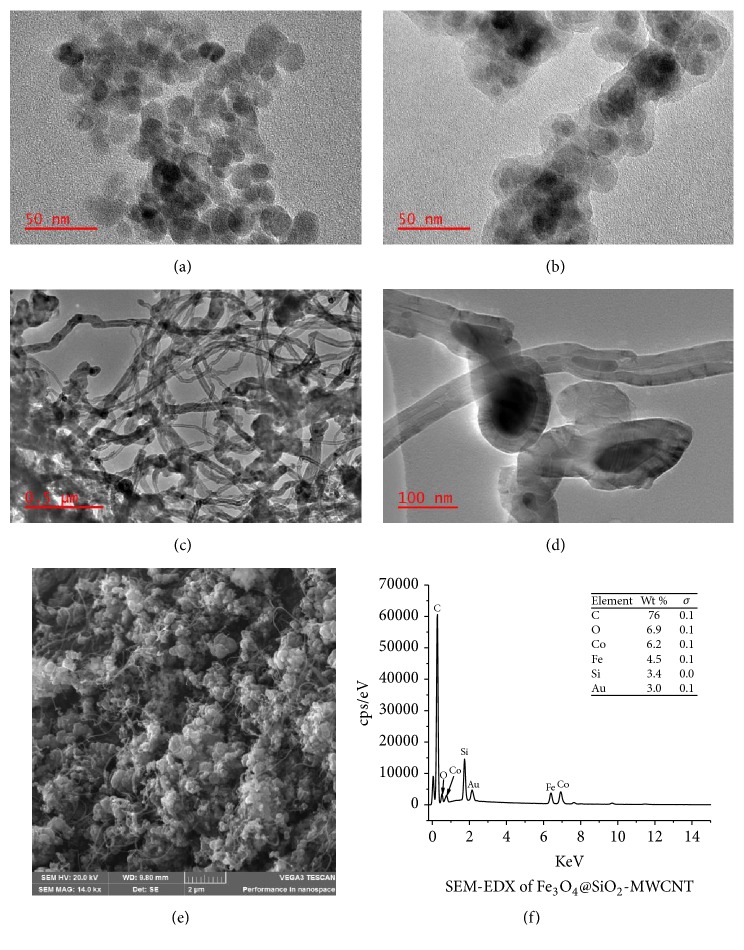
TEM image of Fe_3_O_4_ (a), Fe_3_O_4_@SiO_2_ (b), Fe_3_O_4_@SiO_2_-MWCNT (c-d), SEM image of Fe_3_O_4_@SiO_2_-MWCNT (e), and SEM-EDX of Fe_3_O_4_@SiO_2_-MWCNT (f).

Structural morphologies of Fe_3_O_4,_ Fe_3_O_4_@SiO_2_ and Fe_3_O_4_@SiO_2_-MWCNT nanoparticles were determined by TEM as illustrated in Figure 1. The prepared Fe_3_O_4_ nanoparticle was found to be spherical in shape with agglomeration due to the high surface charge of the Fe_3_O_4_ nanoparticles and magneto dipole interaction [31].  Figure 1(b) shows that the silica-coating process of Fe_3_O_4_ nanoparticle led to the formation of magnetic/silica composite particles with typical core-shell structure. These observations confirmed the formation Fe_3_O_4_@SiO_2_ nanocomposite. On further synthesis of Fe_3_O_4_@SiO_2_-MWCNT nanocomposite using CVD technique, the TEM images in Figures 1(c) and 1(d) show MWCNT with an entangled and disorderly wavy morphology. Both bamboo-like and hollow tube morphology MWCNT were produced during the synthesis [32, 33]. The presence of catalysts encapsulated inside the MWCNT was observed, which were formed as a result of unreacted catalyst particles during the growing process.

The synthesized MWCNT on Fe_3_O_4_@SiO_2_ nanocomposite had an average inner diameter and outer diameter of 9.61±3.68 nm and 30.18±9.7 nm, respectively. The outer diameter of the synthesized MWCNT varied due to differences in particle size and agglomeration of cobalt oxide catalyst [34]. The Fe_3_O_4_ coated with silica (Fe_3_O_4_@SiO_2_) was retained as can be seen in Figure 1(d). This indicates that Fe_3_O_4_ nanoparticle did not participate in the synthesis of MWCNT, hence maintaining and giving the MWCNT nanocomposite the desired magnetic properties.

SEM image of the synthesized Fe_3_O_4_@SiO_2_-MWCNT (Figure 1(e)) shows the surface morphology growth of MWCNT on Fe_3_O_4_@SiO_2._ The highly agglomerated nature of the nanoparticles was observed due to the magnetic properties of the Fe_3_O_4_ in the core shell of the substrate Fe_3_O_4_@SiO_2_. Moreover, the SEM-EDX spectrum shows the peaks of C, Fe, Si, and catalyst used, which confirms the growth of carbon on the surface of the substrate. The Au peak is due to the gold used to coat of the Fe_3_O_4_@SiO_2_-MWCNT nanocomposite before SEM analysis. ICP-OES analysis of the acid digested samples also confirmed the samples consisted of Fe (~4.1 wt. %), Si (~5.9 wt. %), Co (~6.7 wt. %), and C (~79.8 wt. %) only.

#### 3.1.2. X-Ray Diffraction (XRD) Analysis

**Figure 2:**
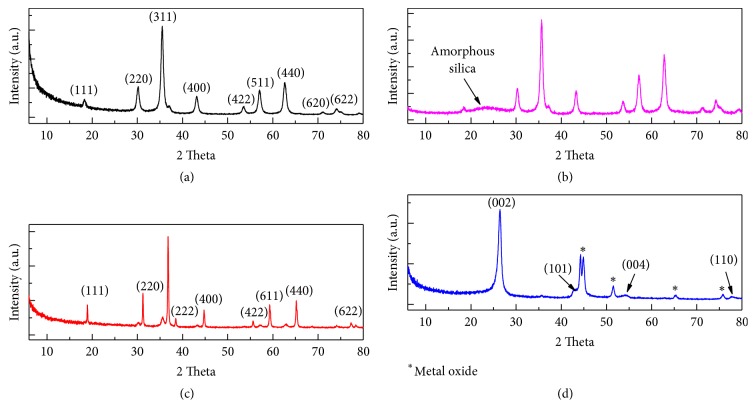
XRD pattern of Fe_3_O_4_ (a), Fe_3_O_4_@SiO_2_ (b), Fe_3_O_4_@SiO_2_-Co (c), and Fe_3_O_4_@SiO_2_-MWCNT (d).

The crystalline structure of Fe_3_O_4,_ nanoparticles before and after silica coating was identified by XRD technique (Figure 2). Fe_3_O_4_ showed a diffraction peaks with 2*θ* at 30°, 35.5°, 43°, 53.5°, 57°, and 62°, which correspond to the crystal planes of (220), (311), (400), (422), (511), and (440), respectively. This indicates a cubic spinel structure of the magnetite [35] and conforms with the reported value of ICDD pdf # 04-006-6497 for Fe_3_O_4_ phase. The diffraction peak 35° (311) was used to calculate the crystalline size of the Fe_3_O_4_ nanoparticle using the Debye-Scherrer formula:

(3) $$\begin{array}{c} D=\frac{K\lambda }{\beta \cos\theta } \end{array}$$

where D is the average crystalline size, *λ* is the X-ray wavelength (0.154nm), *β* is the corrected width of the XRD peak at full width at half maximum (FWHM), and K is a shape factor, which is approximated as 0.9 for magnetite [36].

The calculated crystalline size of Fe_3_O_4_ was estimated to be 12.5 nm. After silica-coating process, Fe_3_O_4_@SiO_2_ nanoparticle was analyzed and indexed using ICDD pdf # 00-063-0731 as illustrated in Figure 2(b). Fe_3_O_4_@SiO_2_ nanoparticle shows a similar diffraction pattern with the Fe_3_O_4_ core except for a broad peak observed between 21° and 27° and a decrease in the intensity of Fe_3_O_4_ nanoparticles_._ This is mainly due to the formation amorphous structure of silica layer on the Fe_3_O_4_ core [37, 38].

Fe_3_O_4_@SiO_2_ was further seeded with cobalt oxide; the XRD peaks for the synthesized Fe_3_O_4_@SiO_2_-Co (Figure 2(c)) were analyzed and indexed using ICDD pdf # 04-015-9577. XRD of Fe_3_O_4_@SiO_2_-Co show characteristic peaks at 2*θ* of 8.9°, 31.8°, 36.6°, 38.3°, 44.5°, 55.3°, 59.0°, 64.8°, and 77.9° which were, respectively, assigned to the indices of (111), (220), (311), (222), (400), (422), (511), (440), and (622). Fe_3_O_4_@SiO_2_-Co nanoparticle was found to be cubic with Fd-3m space group. The seeding of Fe_3_O_4_@SiO_2_ with cobalt oxide catalyst led to the suppression of the amorphous silica peak in Fe_3_O_4_@SiO_2_. This indicates that the Fe_3_O_4_@SiO_2_ was coated with the catalyst and hence the stronger signal from the cobalt oxide. After CVD synthesis, the XRD pattern for the synthesized Fe_3_O_4_@SiO_2_-MWCNT (Figure 2(d)) was analyzed and indexed using ICDD pdf #.04-015-2406. The characteristic peaks at 2*θ* for 26° and 44° were assigned as (002) and (101) of hexagonal graphitic carbon plane of carbon nanotube, respectively. The peak at 26° (002) indicates a relatively high crystalline dimension of graphitic carbon in the synthesized MWCNT on the Fe_3_O_4_@SiO_2_ nanoparticle [39]. Two peaks at approximately 54° (004) and 77° (110) were also observed indicating the high crystallinity of MWCNT. The peaks have been reported in the literature to occur after CVD growth of MWCNT at high temperature [26, 40]. The other peaks with relatively low intensity (indicated with “*∗*” in Figure 2(d)) were for the Fe_3_O_4_ nanoparticle and cobalt oxide catalyst used in the synthesis of Fe_3_O_4_@SiO_2_-MWCNT. The amount of substrate used for CVD growth was 0.2 g, and the amount of MWCNTs grown was approximately 1 g; thus it was expected that the intensity of the metal oxide peaks would be lower than the graphitic MWCNT peaks, due to the smaller amount of metal oxide present in the Fe_3_O_4_@SiO_2_-MWCNT nanocomposite.

#### 3.1.3. Textural Characteristics of the Nanomaterials and Nanocomposite

**Figure 3:**
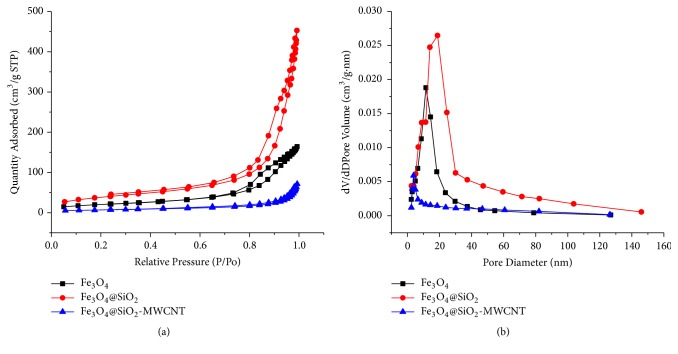
Panel (a) is the nitrogen adsorption-desorption isotherm for Fe_3_O_4_@SiO_2_-MWCNT, and panel (b) is the corresponding PSD.

The textural characteristics were determined from nitrogen adsorption-desorption isotherms, and the isotherms and pore size distributions (PSDs) for Fe_3_O_4_, Fe_3_O_4_@SiO_2_ and Fe_3_O_4_@SiO_2_-MWCNT are illustrated in Figure 3. The isotherms were classified according to the IUPAC system as type IV. Fe_3_O_4,_ Fe_3_O_4_@SiO_2_ and Fe_3_O_4_@SiO_2_-MWCNT had a hysteresis loop of H1, which is often associated with materials that agglomerates or compacts of approximately spherical particles arranged in a fairly uniform manner. It is also observed with materials with cylindrical pore geometry and uniform pore sizes [41, 42].

The BET surface area of Fe_3_O_4,_ Fe_3_O_4_@SiO_2_ and Fe_3_O_4_@SiO_2_-MWCNT was 73, 139, and 25 m^2^/g, respectively. It was observed that after coating Fe_3_O_4_ with SiO_2_ there was an increase in specific surface area and pore volume of the silica iron oxide composite when compared to the iron oxide nanoparticles. This can be attributed to the silica coating, which seems to have reduced the agglomeration of the iron oxide nanoparticles and increased the size of the interparticles voids in the powder (see increased PSD on Figure 3(b)). However, after CVD synthesis there was a significant decrease in the surface area of the resulting nanocomposite. From the TEM analysis, the MWCNT tips were capped with carbon and blocked with catalysts particles, thus the inner core of the CNTs does not contribute to the surface area, and this may explain the decrease in surface area.

The average pore diameters for Fe_3_O_4,_ Fe_3_O_4_@SiO_2_, and Fe_3_O_4_@SiO_2_-MWCNT were found to be 12.0 nm, 18.8 nm, and 3.7 nm, respectively, from desorption branch of the isotherm, using the BJH (Barett-Joyner-Halenda) method of analysis. The porosity observed is due to the voids between individual nanoparticles. Thus, an increase in the pore diameter from the iron oxide nanoparticles to the Fe_3_O_4_@SiO_2_ nanocomposite indicates the silica coating has aided in preventing some agglomeration between the magnetic nanoparticles and thus increasing the separation between individual nanoparticles. The lower average pore diameter of Fe_3_O_4_@SiO_2_-MWCNT is a result of the highly entangled network of MWCNTs. The Fe_3_O_4_@SiO_2_-MWCNT nanocomposite has a low surface area and low PSD values which indicates the tips of the nanotubes are capped and not open (as observed in TEM analysis). Hence, the inner cavity of the nanotubes is not easily accessible and contributes very little to the surface area or PSD values. Furthermore, the low PSD and surface area indicate a highly entangled web of nanotubes with the Fe_3_O_4_@SiO_2_-MWCNT nanocomposite, which correlates with the SEM and TEM observations.

#### 3.1.4. Raman Spectroscopy Investigations

The defect and crystallinity of MWCNT were evaluated using Raman spectroscopy as illustrated in Figure S4. Raman spectra for Fe_3_O_4_@SiO_2_-MWCNT showed D, G, and G' band with additional peaks at 291, 481, and 667 cm^−1^, respectively, due to the Fe–O bond [43]. The G band has *E*_2g_ symmetry, which arises from in-plane bond stretching mode of the C-C bond, reflecting the structural intensity of the sp^2^ hybridized carbon atoms. Meanwhile, the D band is a symmetrical stretch with *A*_1g_ symmetry, which relates to the degree of disorder in carbon sp^2^ bonded clusters in graphite and tube end. The G' band is indicative of long-range order in a sample and arises from the two-phonon, second-order scattering process that results in the creation of an inelastic phonon [44–47].

The integrated intensity ratio I_D_/I_G_ for the D and G bands was used to measure the defect in Fe_3_O_4_@SiO_2_-MWCNT. A higher intensity ratio of I_D_/I_G_ indicates more defects present inside the carbon layers of MWCNTs. Thus, I_D_/I_G_ ratio of the Fe_3_O_4_@SiO_2_-MWCNT and acid treated Fe_3_O_4_@SiO_2_-MWCNT was 0.81 and 0.93, respectively, suggesting that there was less structural defect during the synthesis of this nanocomposite [48]. The increase in ID/IG does suggest introduction of defect sites onto the CNTs, which indicates the acid treatment altered the CNTs and may have improved the sorption properties of the CNT layer on the nanocomposite.

### 3.2. Extraction Study

The quantitative extraction of azinphos methyl, chlorpyrifos, malathion, and parathion mixture using the synthesized Fe_3_O_4_@SiO_2_@MWCNT nanocomposite was performed. Various factors, which influence the extraction recovery of the selected pesticides like adsorbent dosage, solution pH, and adsorption time, were optimized using CCD.

#### 3.2.1. Optimization of OPPs Using RSM

The experimental design was used to optimize the parameters that may affect the extraction performance of OPPs from water using the synthesized Fe_3_O_4_@SiO_2_-MWCNT in order to obtain the best possible extraction condition for our synthesized material. Thus, using response surface methodology (RSM) design of experiments (DoE), specifically, central composite design (CCD) in Statistica version 8 (StatSoft, USA), Table S2 was generated. By adopting the central composite design, it was thus possible to evaluate the effects of pH (X_1_), adsorbent dosage (X_2_), and time (X_3_) on extraction efficiency. The results obtained from the experimental runs are presented in Table 1.

**Figure 4:**
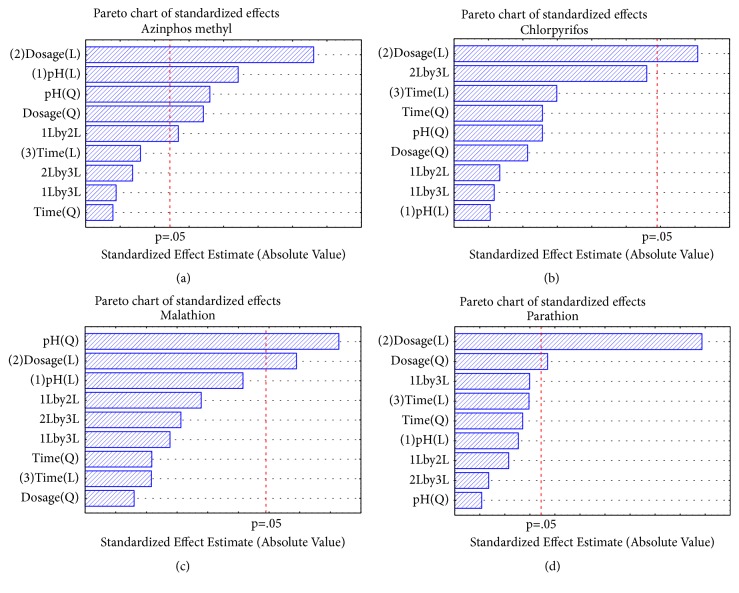
Pareto chart of standardized effects showing the p-value of azinphos methyl (a), chlorpyrifos (b), malathion (c), and parathion (d).

Second-degree polynomial multiregression model was fitted to the experimental data and the resultant model fit described the linear and quadratic effects of the variables by means of analysis of variance (ANOVA) as shown in the Pareto chart in Figure 4. The p-values were used as a tool to check the significance of each of the coefficients, which in turn are necessary to understand the pattern of the mutual interactions between the test variables. A p-value less than 0.05 in the ANOVA indicates the statistical significance of an effect at 95% confidence level [30, 49]. The reference line indicated on the Pareto chart (*α* = 0.05) distinguishes between significant and insignificant effects, such that any effect that extends beyond this reference line is significant, whereas linear effect (L) of a variable means that the variable correlates directly proportional to the response variable, whereas the quadratic effect (Q) of a variable implies that the response variable is correlated with the square of that variable [50].

In this study, it was observed that adsorbent dosage had a significant effect (p<0.05) in the extraction of all the selected OPPs, while the pH of the solution only significantly affected azinphos methyl and malathion. On the other hand, extraction time did not have a significant effect (p>0.05) on the extraction of the OPPs (Figure 4).

**Figure 5:**
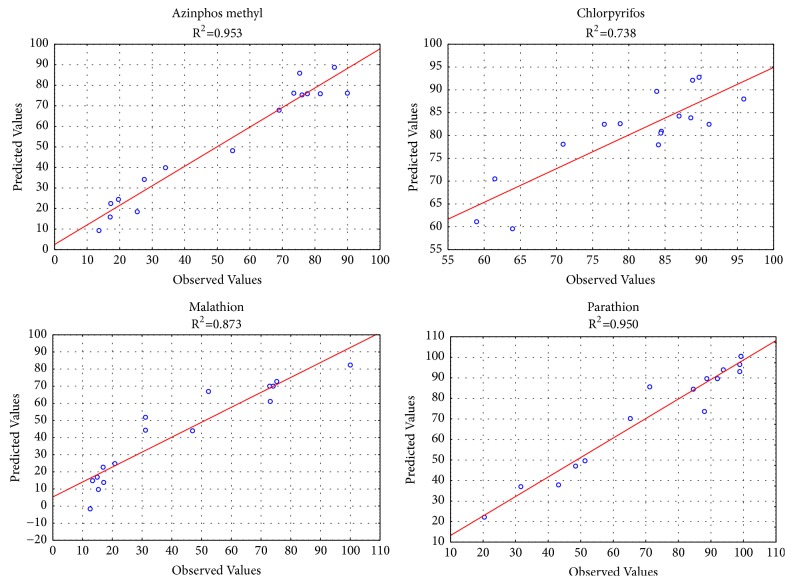
The experimental data vs. the predicted data of normalized probability results extraction recovery (ER %) of azinphos methyl (a), chlorpyrifos (b), malathion (c), and parathion (d).

The plot of experimental values of extraction recovery (ER%) versus those calculated from equation (1) indicated a good fit, as presented in Figure 5. The fits of the polynomial model were also expressed by the coefficient of determination (R^2^), which was found to be 0.953, 0.738, 0.873, and 0.950 for azinphos methyl, chlorpyrifos, malathion, and parathion, respectively, meaning that 95, 74, 87, and 95% of the variability in the response could be explained by the model (Figure 5). This is also evident from the fact that the plot of predicted versus experimental values of OPPs correlation coefficient is close to y=x, showing that the prediction of experiment is quite satisfactory.

#### 3.2.2. Response Surface Methodology

**Figure 6:**
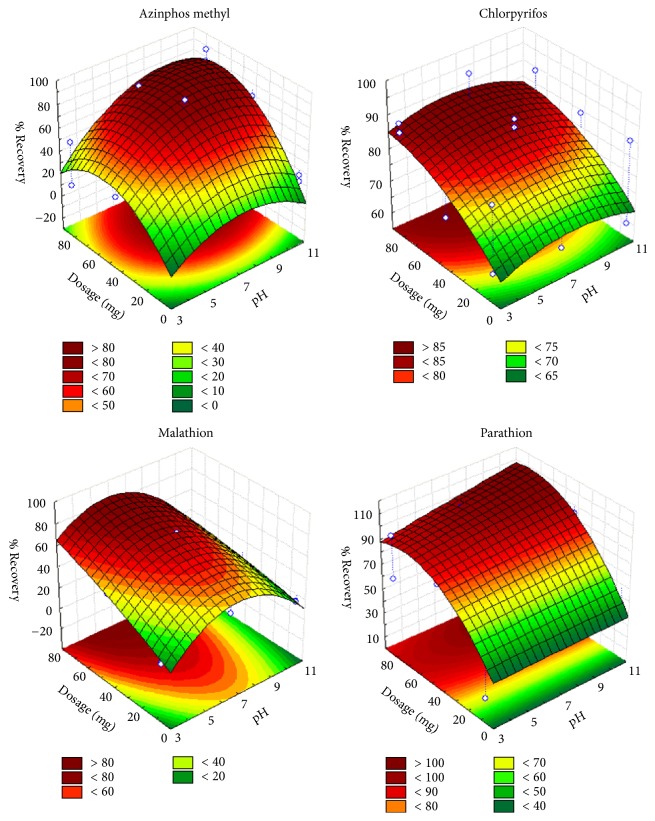
Surface response generated from a quadratic model in the optimization of pH and adsorbent dosage for azinphos methyl, chlorpyrifos, malathion, and parathion.

Response surface methodology (RSM) was developed by considering all the significant interactions in the CCD to optimize the critical factors and describe the nature of the response surface in the experiment. Three-dimensional surface plots were generated from the model fit in order to visually describe the interrelationship between the levels of factors and the recovery patterns of the OPPs (Figure 6). These plots were obtained for a given pair of factors at fixed and optimal values of other variables. The obtained curves of the plots indicate that there was interaction between the variables.

**Figure 7:**
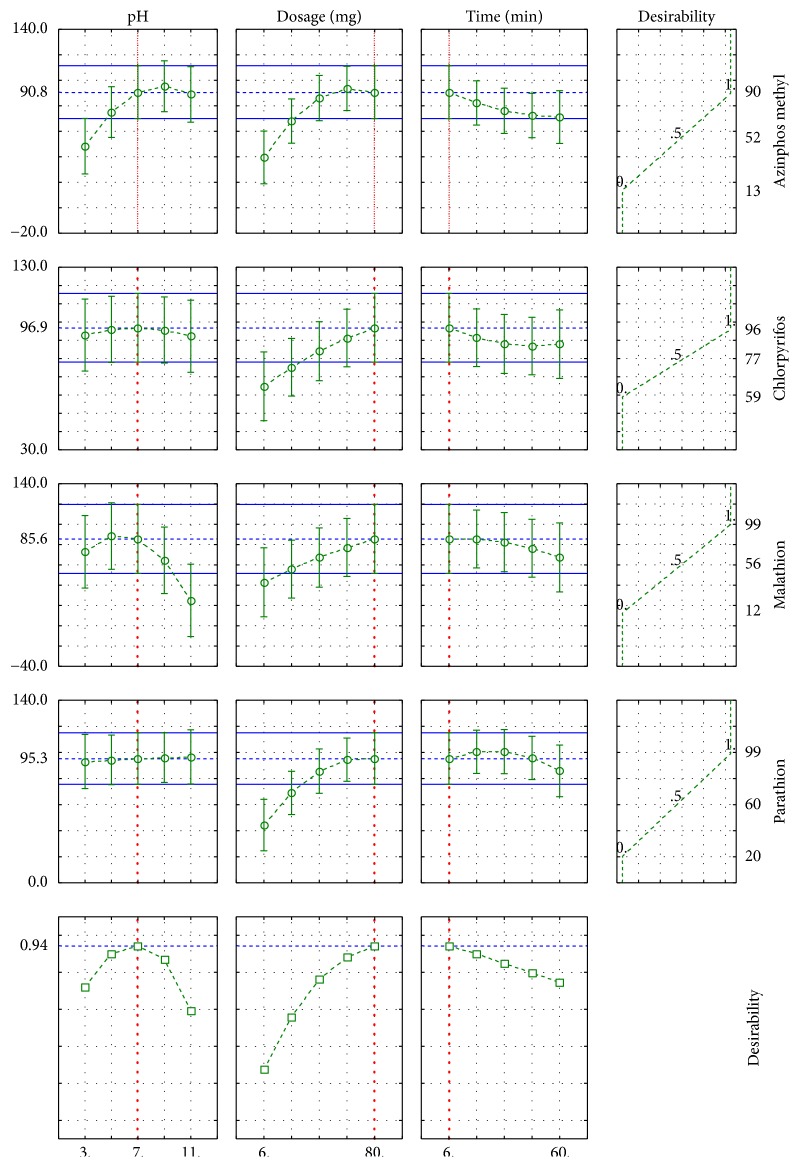
Profiles for predicated values and desirability function for extraction of azinphos methyl, chlorpyrifos, malathion, and parathion mix. Dashed line indicated current values after optimization.

From the model fit, it is observed that there is an increase in extraction recovery as the adsorbent dosage increases, which is due to the increased number of adsorption sites. The pH of a solution is also an important parameter affecting both the charge and stability in the extraction of OPPs during the adsorption process. It was observed that the pH of the solution had a significant influence in the extraction recovery of OPPs during extraction when using Fe_3_O_4_@SiO_2_-MWCNT adsorbent. High recovery of OPPs was observed at pH of 7. Chlorpyrifos and parathion were not affected by the pH of the solution (Figures 6 and 7) whereas malathion and azinphos methyl were significantly affected by pH of the solution. This could be due to the fact that malathion and azinphos methyl can decompose within acidic and alkaline solution, and this can affect the recoveries. In addition, at low pH there was a decrease in extraction recovery of azinphos methyl by the Fe_3_O_4_@SiO_2_-MWCNT adsorbent. At low pH values the functional groups of Fe_3_O_4_@SiO_2_-MWCNT surface are protonated, and since azinphos methyl is a weak acid (pKa = 5) it is most likely protonated at low pH values, and therefore there will be relatively greater repulsive forces between the absorbent and absorbate at low pH values. At higher pH values, the delocalized system on the azinphos methyl most likely favours *π*-*π* stacking and/or electrostatic interactions between the adsorbent and the substrate, thus improving recoveries. In contrast, malathion has poor recoveries at high pH; besides the possibility of degradation, electrostatic repulsions (negatively charged MWCNT surface) and a lack of *π*-*π* stacking interactions (no delocalized ring on malathion vs. azinphos methyl, chlorpyrifos, and parathion) at high pH may contribute to the lower recoveries.

#### 3.2.3. Optimization of CCD by Desirability Function (DF) for Extraction Procedure

Since our extraction procedure involved simultaneous extraction of all the four OPPs, we considered a global optimization approach, which would establish the best extraction condition for all of the pollutants. In this regard, the desirability function built into Statistica 8.0 software was a useful tool. Profiling the desirability of responses involved specifying the DF for each dependent variable (% extraction recovery) by assigning predicted values. This was done by using scale in the range of 0.0 (undesirable) to 1.0 (very desirable) for the selection and optimization of designed variables. DF value for each dependent variable in Figure 7 shows that desirability of 1.0 was assigned to maximum %ER (89.8, 95.9, 99.8, and 99.2%) and 0.0 for minimum %ER (13.6, 58.9, 12.5, and 20.2%), and 0.5 value was assigned to represent the middle %ER (51.8, 77.4, 56.1, and 59.7%) for azinphos methyl, chlorpyrifos, malathion, and parathion, respectively. The individual desirability value calculated for the percentage extraction recovery of the OPPs mix which was found to be 0.94 (94%) (close to 1.0) as illustrated in Figure 7 (*bottom left*) was obtained. Since desirability of 1.0 was selected as the target value with maximum recovery of 89.8, 95.9, 99.8, and 99.2% for azinphos methyl, chlorpyrifos, malathion, and parathion, respectively, the optimum extraction conditions were set at 80 mg of adsorbent dosage, pH of 7 with a contact time of 6 min.

### 3.3. Evaluation of the Method Performance

The method was evaluated by determining the linear range, coefficient of determination (R^2^), RSD, LOD, and LOQ under optimum conditions. The external calibration curve was established using six different concentrations of the OPPs standard solution. The quantification ions were used in the calculation of the absolute peak area. The characteristic calibration data obtained are summarized in Table 2. Good linearity was observed over the wide concentration ranges for the OPPs with satisfactory R^2^. The LOD and LOQ were determined based on the signal to noise ratio of 3 and 10, respectively [51–53]. From the results summarized in Table 2, the LOD for the OPPs were found to be in the range of 0.004-0.150 *μ*g/L, whereas LOQs ranged from 0.013 to 0.499 *μ*g/L.

To assess the precision of the method, a repeatability study was conducted by analyzing 5 parallel experiments of water samples obtained from Vaal Dam by spiking OPPs mix with 50 *μ*g/L (azinphos methyl, chlorpyrifos, parathion, and malathion) under optimum conditions. The RSD% results for azinphos methyl, chlorpyrifos, parathion, and malathion pesticides were in the range of 3.6-5.7% (n=5), indicating that the repeatability of the current method was within the acceptable range stipulated by the U.S. Environmental Protection Agency (EPA), which should have maximum RSD% of 30% [54]. The enrichment factor (EF), which is defined as the ratio of slope of calibration obtained from the proposed extraction method to that without preconcentration, was in the range between 394 and 721 (Table 2). These values were higher than magnetic solid phase extraction of OPPs done by using Fe_3_O_4_@SiO_2_–C18 nanoparticles [55] as well as Fe_3_O_4_/CNT nanoparticles [56] indicating that Fe_3_O_4_@SiO_2_-MWCNT nanocomposite exhibited a high adsorption capacity for the target analytes. This could be ascribed to the silica layer and the functionalized MWCNTs on the nanocomposite.

### 3.4. Application on Environmental Samples

To assess the applicability of the proposed MSPE technique using Fe_3_O_4_@SiO_2_-MWCNT as an adsorbent, OPPs were extracted from environmental water samples collected from Vaal River and Vaal Dam. The samples were analyzed by using the optimum conditions obtained.  Figure S5 shows the HPLC chromatograms obtained following the preconcentration of spikes water samples on MSPE under optimized conditions. Azinphos methyl, chlorpyrifos, parathion, and malathion were detected in both Vaal River and Vaal Dam (Table 3) except for parathion, which was only recovered from Vaal River. Chlorpyrifos levels were high in both Vaal River and Vaal Dam due to run-offs from agricultural fields along the river confirming that, among the four OPPs, chlorpyrifos is the most extensively used pesticide in the area. This was also observed by Dabrowski and coworkers [57] when they determined the presence of OPPs in the Lourens River, Cape Town.

Hence, to verify the applicability of the method, river water and dam water were spiked with known concentration of the OPPs mix and then analyzed to determine the recoveries of the method. Nonspiked Vaal River and Vaal Dam water sample and blanks were also analyzed to evaluate contamination resulting from the complete preparation and analytical procedure and subtracted from spiked sample to determine absolute recoveries. For each concentration, three parallel experiments were performed. The resultant recoveries of analytes following this extraction method were in the range of 84 to 101.4% (% RSDs range of 2.9 to 10.4%) as summarized in Table 3. The data generated reveal that the method is suitable for the extraction and analysis of OPPs in environmental water sample.

### 3.5. Recycling and Reuse of the Adsorbent

**Figure 8:**
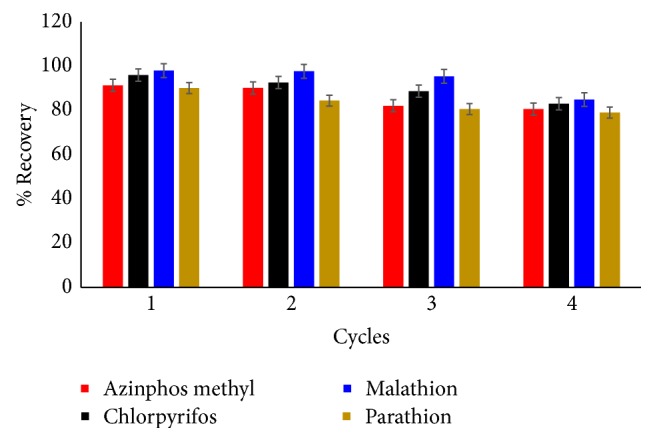
Recycling and reuse of the Fe_3_O_4_@SiO_2_@MWCNT adsorbent in the removal of azinphos methyl, chlorpyrifos, malathion, and parathion mix from aqueous solution.

In order to examine the reusability of the Fe_3_O_4_@SiO_2_-MWCNT adsorbent, the used adsorbent was washed twice with 2 mL of acetone, and then with 2 mL of distilled water by vortexing for approximately 2 min. The adsorbent was magnetically collected and reused for the next analysis run. In each analysis run, 50 *μ*g/L of OPPs sample solution was tested according to the procedure previously described in the experimental section under optimal conditions. The percentage recovery of the azinphos methyl, dimethoate, chlorpyrifos, parathion, and malathion pesticides after five cycles of adsorption-desorption process was in the range of 84-94% (Figure 8). Thus, this indicates that the synthesized Fe_3_O_4_@SiO_2_-MWCNT nanocomposite could be reused at least 5 times without a significant loss in its adsorption capacity. Therefore, the proposed method possesses acceptable reusability.

### 3.6. Comparison of Extraction of Fe_*3*_O_*4*_@SiO_*2*_-MWCNT with Literature

A comparison of the newly synthesized Fe_3_O_4_@SiO_2_-MWCNT in the extraction of OPPs in water with previously reported materials [55, 58–60] is summarized in Table 4. The data indicates that a wide variety of materials have been applied for the removal of pesticides using different types of materials. The present study has a short extraction time at a pH of 7. Thus, there was no need to adjust the sample solution pH.

The reason for the short extraction time with the Fe_3_O_4_@SiO_2_-MWCNT nanocomposite is unclear. Carbon nanomaterials can sorb organic molecules via *π*-*π*, hydrophobic, hydrophilic, dispersion forces, or electrostatic interactions [61]. However, the key differences with the Fe_3_O_4_@SiO_2_-MWCNT nanocomposite include the large amount of MWCNTs, the excellent mesoporous network from the entangled web of nanotubes, and the average outer diameter of the MWCNTs. These factors may play a role in the shorter extraction time observed. Furthermore, there may be some synergy from the silica support. Overall, the method also showed good reusability of the sorbent, satisfactory %RSD, and good recoveries revealing that the proposed method for the analysis of OPPs in water sample is rapid, simple, precise, and sensitive.

## 4. Conclusions

In this study, MWCNTs were successfully synthesized on magnetic silica (Fe_3_O_4_@SiO_2_) substrate using cobalt oxide as a catalyst and acetylene gas as carbon source. The TEM observation clearly showed an iron oxide core, a separate silica layer coating the core, and entangled tubular structures of MWCNTs on the surface. The XRD analysis confirmed the separate phases of iron oxide, silica, and the coating of the metal catalyst before CVD growth of the MWCNTs. From the TEM and XRD investigations, the nanocomposite retained the magnetic iron oxide core, protected by the silica layer, and a layer of MWCNTs. Using response surface methodology, the optimum conditions for the simultaneous extraction of four pesticides from aqueous media were determined. A maximum extraction recovery of 89.8, 95.9, 99.8, and 99.2% for azinphos methyl, chlorpyrifos, malathion, and parathion, respectively, at optimum conditions of 80 mg of adsorbent dosage, pH of 7 with a contact time of 6 min, was obtained. High recovery of OPPs was attributed to large delocalized *π*-electron system on the surfaces of MWCNT, which plays the main role in *π*-stacking interactions with the aromatic rings of OPPs or their electronegative atoms (P, N, and S). The Fe_3_O_4_@SiO_2_-MWCNT nanocomposite also showed fast magnetic separation from sample solution, good reusability of the sorbent, and relatively quick extraction time (about 6 min). The presence of azinphos methyl, chlorpyrifos, parathion, and malathion pesticides was found in both Vaal River and Vaal Dam indicating there was either point or nonpoint source pollution of the environment water. Therefore, the synthesized Fe_3_O_4_@SiO_2_-MWCNT nanocomposite has a potential for the extraction of OPPs from water samples. Further work on simultaneous extraction of emerging pollutants and pesticides from environmental samples is on-going.

## Figures and Tables

**Table 1:** Percentage extraction recovery of azinphos methyl, chlorpyrifos, parathion, and malathion using Fe_3_O_4_@SiO_2_-MWCNT adsorbent.

pH	Adsorbent dosage (mg)	Adsorbent time (min)	% Extraction recovery			
			Azinphos methyl	Chlorpyrifos	Malathion	Parathion
3	80	6	55	90	75	99
3	43	33	34	71	47	85
7	43	6	75	89	52	71
3	6	6	17	64	17	51
11	6	60	20	84	13	31
7	43	60	82	84	73	88
7	43	33	90	91	73	92
11	6	6	25	59	12	43
7	43	33	73	77	74	89
11	80	60	76	79	15	99
3	80	60	17	87	31	65
7	6	33	27	61	31	48
3	6	60	14	84	15	20
11	43	33	69	84	17	94
7	80	33	78	96	100	99
11	80	6	86	89	21	99

**Table 2:** The analytical performance of MSPE-LC-MS method for determination of OPPs.

OPPs	Regression equation	R^2^	Linear range (*μ*g/L)	LOD (*μ*g/L)	LOQ (*μ*g/L)	RSD (%, n=5)	EF
Azinphos methyl	y = 2864.6x + 14251	0.9977	10-200	0.004	0.013	5.7	721
Chlorpyrifos	y = 488.28x + 870.33	0.9976	10-200	0.026	0.085	3.6	541
Malathion	y = 2203x - 1349	0.9971	10-200	0.006	0.021	5.1	394
Parathion	y = 113.78x + 85.255	0.9955	10-200	0.150	0.499	4.3	431

**Table 3:** Determination of OPPs in water sample (nd = not detected and D = detected).

OPPs	Vaal River water				Vaal Dam water		
	spiked (*μ*g/L)	Quantified (*μ*g/L)	%R(a)	RSD %	Quantified (*μ*g/L)	%R(a)	RSD %
Azinphos methyl	0	D			D		
	10	9.1	90.8	3.8	9.4	93.6	6.5
	50	45.2	90.4	6.2	46.3	92.6	4.9
Chlorpyrifos	0	D			D		
	10	8.6	86.0	5.9	9.1	91.5	10.4
	50	46.7	93.5	5.6	46.9	93.8	5.5
Malathion	0	D			D		
	10	8.8	88.3	6.3	8.6	86.3	7.5
	50	42.0	84.0	9.6	43.1	86.2	2.9
Parathion	0	nd			D		
	10	10.1	101.4	4.8	8.9	88.7	5.9
	50	45.8	91.5	8.2	45.1	90.2	9.8

**Table 4:** Comparison of current work for pesticide extraction with reported literature materials.

Adsorbent	Method	AD	n	CT	pH	% RSD	% R	Ref
MWCNT	DSPE-GC-NPD	100-500	15	30	Neutral range	<10.1%	67-107%	[58]
C18-SiO_2_-Fe_3_O_4_	MSPE-HPLC-UV	50	2	10	6	5.4-7.2%	85-92%	[59]
Fe_3_O_4_@SiO_2_–C18	MSPE-HPLC-UV	70	2	10	6	9.2-9.8%	86-90%	[55]
Fe_3_O_4_@CNT	MSPE–HPLC	51.84	3	36	11.15	2.3-4.5%	60-92%	[60]
Fe_3_O_4_@C	MSPE-HPLC-UV	60	3	30	4	2.7-4.5 %	60-92%	[5]
G-CNT-Fe_3_O_4_	MSPE/HPLC–UV	80	4	15	4 to 8	3.9-8.8	75-102%	[62]
Fe_3_O_4_@SiO_2_-MWCNT	MSPE-LC-MS	80	4	6	7	2.9-10.4%	84-101%	This method

n=number of analytes; AD=adsorbent dosage (mg), CT = contact time (min), % R =% recovery, and % RSD=% relative standard deviation.

## Data Availability

The data used to support the findings of this study are included within the article and within the supplementary information file.

## Supplementary Materials

Supplementary Materials The supplementary materials consist of Figures S1–S5 and Tables S1–S2. Figure S1 provides the molecular structures of the organophosphorus pesticides that were used in this study. Figure S2 is a simple schematic of the synthesis of the magnetic nanocomposite Fe3O4@SiO2 and Figure S3 shows an outline of the CVD process used to coat MWCNTs onto the magnetic nanocomposite. Figure S4 provides the Raman spectra for the Fe3O4@SiO2-MWCNT nanocomposite. Figure S5 is a sample HPLC total ion chromatogram of an environmental water sample spiked with the organophosphorus pesticides. The MRM transitions, MS conditions, and retention times of the organophosphorus pesticides and the design of experiment factors and levels are presented in Tables S1 and S2, respectively.
