# Supplementary material for: Chemical Vapour Deposition of MWCNT on Silica Coated Fe3O4 and Use of Response Surface Methodology for Optimizing the Extraction of Organophosphorus Pesticides from Water
Source: Int J Anal Chem. 2019 Jul 3;2019:4564709. doi: 10.1155/2019/4564709 (PMC6636531; doi:10.1155/2019/4564709)
Supplement: Supplementary Materials — The supplementary materials consist of Figures S1–S5 and Tables S1–S2. Figure S1 provides the molecular structures of the organophosphorus pesticides that were used in this study. Figure S2 is a simple schematic of the synthesis of the magnetic nanocomposite Fe3O4@SiO2 and Figure S3 shows an outline of the CVD process used to coat MWCNTs onto the magnetic nanocomposite. Figure S4 provides the Raman spectra for the Fe3O4@SiO2-MWCNT nanocomposite. Figure S5 is a sample HPLC total ion chromatogram of an environmental water sample spiked with the organophosphorus pesticides. The MRM transitions, MS conditions, and retention times of the organophosphorus pesticides and the design of experiment factors and levels are presented in Tables S1 and S2, respectively. [file 4564709.f1.docx]

**Chemical vapour deposition of MWCNT on silica coated Fe_3_O_4_ and use of response surface methodology for optimizing the extraction of organophosphorus pesticides from water**

Veronica W. O. Wanjeri^1^, Sefater Gbashi^2^, Jane C. Ngila^1^, Patrick Njobeh^2^, Messai A. Mamo^1^, and Patrick G. Ndungu^1^*

^1^*University of Johannesburg, Department of Chemical Science,* *Energy, Sensors and Multifunctional Nanomaterials Research Group, P.O Box 17011 Doornfontein 2028, South Africa*

^2^ *Department of Biotechnology and Food Technology, University of Johannesburg South Africa*

Corresponding author: Ndungu, P.G; Email; pndungu@uj.ac.za; Tel: +27115596180

**Supplementary Information**

Figure S1: Chemical structures of selected OPPs used in the study.

Figure S2: Schematic for the synthesis of Fe_3_O_4_@SiO_2._

Figure S3: Schematic diagram of apparatus used for CNT synthesis.

Table S1: MRM transitions, MS conditions and retention times of the determined OPPs

| **Name of analyte** | **Precursor (m/z)** | **Quantifier product ion (m/z)** | **Qualifier product ion (m/z)** | **Q1 pre-bias (V)** | **Collision energy (eV)** | **Q3 pre-bias (V)** | **Retention time (min)** |
| --- | --- | --- | --- | --- | --- | --- | --- |
| **Azinphos methyl** | 317.90 | 132.05 | 159.90 | -16, -16 | -17, -8 | -29, -28 | 5.41 |
| **Chlorpyrifos** | 351.50 | 96.95 | 125.00 | -17, -17 | -37, -20 | -15, -21 | 6.56 |
| **Malathion** | 330.90 | 127.05 | 284.80 | -17, -12 | -13, -9 | -21, -29 | 5.71 |
| **Parathion** | 291.90 | 94.15 | 123.05 | -14, -15 | -40, -35 | -15, -26 | 5.99 |

Table S2: Design of experiment (factors and levels), and the range and levels used

| **Pattern** | **pH** | **Time (min)** | **Adsorbent Dosage (mg)** |
| --- | --- | --- | --- |
| 0 | 0 | 0 | 0 |
| +−+ | 1 | -1 | 1 |
| 0a0 | 0 | -1 | 0 |
| ++− | 1 | 1 | -1 |
| 0A0 | 0 | 1 | 0 |
| −++ | -1 | 1 | 1 |
| +++ | 1 | 1 | 1 |
| A00 | 1 | 0 | 0 |
| 00A | 0 | 0 | 1 |
| a00 | -1 | 0 | 0 |
| −−+ | -1 | -1 | 1 |
| −−− | -1 | -1 | -1 |
| 0 | 0 | 0 | 0 |
| −+− | -1 | 1 | -1 |
| 00a | 0 | 0 | -1 |
| +−− | 1 | -1 | -1 |
|  | **Range and level** | | |
|  | **Low (-1)** | **Middle (0)** | **High (+1)** |
| **Dosage (mg)** | 6 | 43 | 80 |
| **Time (min)** | 6 | 33 | 60 |
| **pH** | 3 | 7 | 11 |

FigureS4: Raman spectra of Fe_3_O_4_@SiO_2_-MWCNT.

**Figure S5: HPLC Total ion chromatogram of Vaal Dam spiked (50 µg/L) with Azinphos methyl, chlorpyrifos, parathion and malathion under optimized conditions.**
